# Generation and characterization of a hypothyroidism rat model with truncated thyroid stimulating hormone receptor

**DOI:** 10.1038/s41598-018-22405-7

**Published:** 2018-03-05

**Authors:** Jianqiang Yang, Ning Yi, Junhui Zhang, Wen He, Di He, Wanwan Wu, Shuyang Xu, Feng Li, Guoping Fan, Xianmin Zhu, Zhigang Xue, Wensheng Zhou

**Affiliations:** 1 Department of Otorhinolaryngology Head and Neck Surgery, The First Affiliated Hospital of Nanchang University, Nanchang, Jiangxi 330006 China; 2 Translational Center for Stem Cell Research, Tongji Hospital, Department of Regenerative Medicine, Tongji University School of Medicine, Shanghai, 200065 China; 3 Shanghai Pulmonary Hospital, School of Life Sciences and Technology, Tongji University, Shanghai, 200433 China; 4 Shanghai Public Health Clinical Center, Fudan University, Shanghai, 201508 China; 5 Department of Human Genetics, David Geffen School of Medicine, University of California Los Angeles, Los Angeles, CA 90095 USA

## Abstract

Thyroid stimulating hormone receptor (TSHR), a G-protein-coupled receptor, is important for thyroid development and growth. In several cases, frameshift and/or nonsense mutations in TSHR were found in the patients with congenital hypothyroidism (CH), however they have not been functionally studied in an animal model. In the present work, we generated a unique *Tshr*^*Df/Df*^ rat model that recapitulates the phenotypes in TSHR Y444X patient by CRISPR/Cas genome editing technology. In this rat model, TSHR is truncated at the second transmembrane domain, leading to CH phenotypes as what was observed in the patients, including dwarf, thyroid aplasia, infertility, TSH resistant as well as low serum thyroid hormone levels. The phenotypes can be reversed, at least partially, by levothyroxine (L-T4) treatment after weaning. The thyroid development is severely impaired in the *Tshr*^*Df/Df*^ rats due to the suppression of the thyroid specific genes, i.e., thyroperoxidase (*Tpo*), thyroglobulin (*Tg*) and sodium iodide symporter (*Nis*), at both mRNA and protein levels. In conclusion, the *Tshr*^*Df/Df*^ rat serves as a brand new genetic model to study CH in human, and will greatly help to shed light into the development of terminal organs that are sensitive to thyroid hormones.

## Introduction

**Figure 1:**
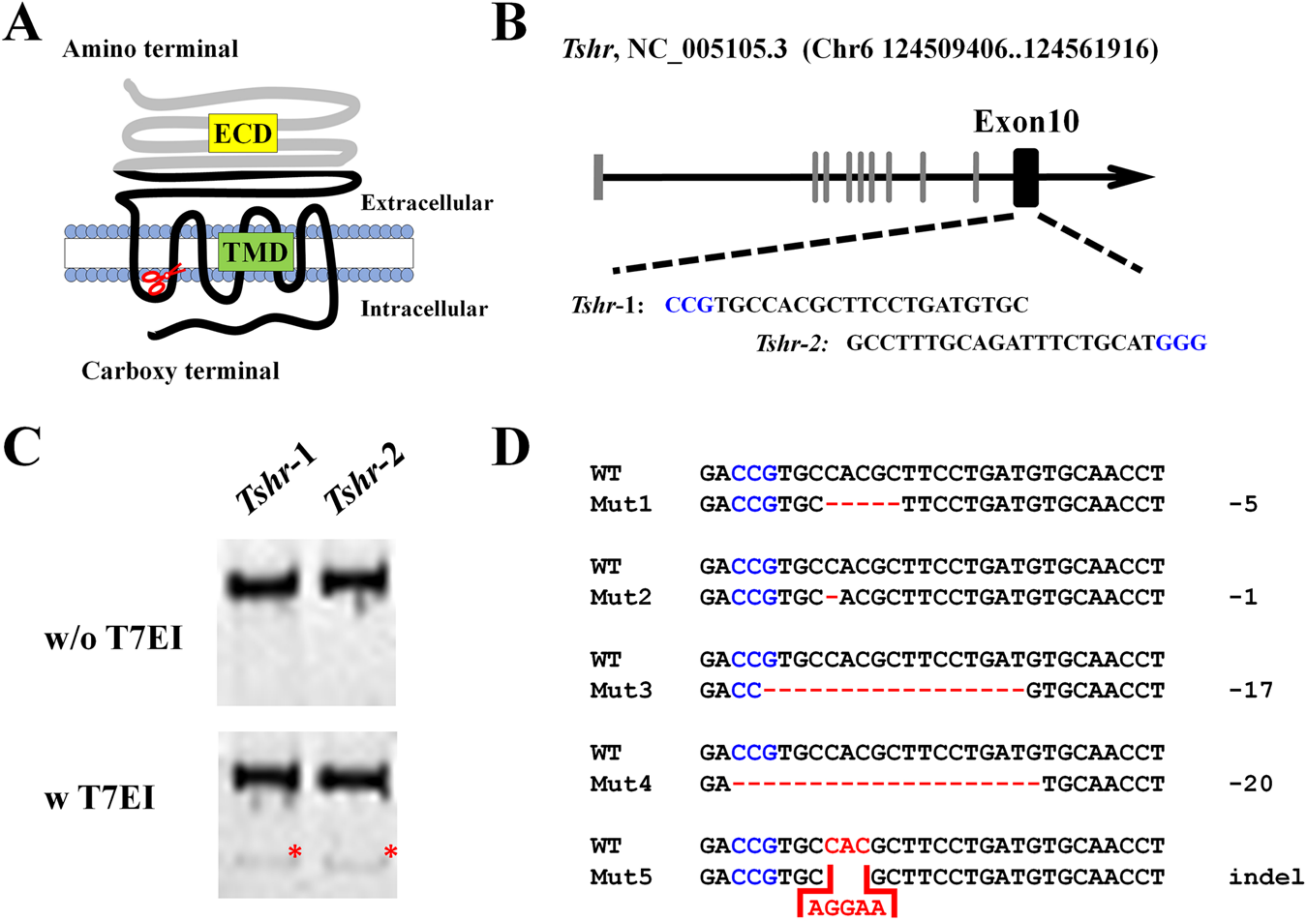
Generation of *Tshr* mutant rats by CRISPR/Cas9 technology. (**A**) The diagram showed the TSHR protein domains, including extracellular domain (ECD), transmembrane domains (TMD), and one intracellular carboxy terminal. The gray line refers to the ECDs encoded by exon 1–9, while the black line refers to the domains encoded by exon 10. The scissor indicates the gRNA targeting sequence near the end of 1^st^ intracellular loop (ICL). (**B**) DNA sequences of the gRNA targeting sites. The sgRNA targeting sequence is shown in black, and the PAM sequence is shown in blue. (**C**) The targeting and cutting efficiency of CRISPR was tested by a T7 endonuclease I (T7EI) assay *in vitro*. The image was cropped from different parts of the same gel. The full-length gel is presented in Supplementary Fig. 3. (**D**) Sequencing results of the mutant alleles in the founders. the PAM squence is highlighted in blue. The deletions are indicated as “−”, whereas the insertions are highlighted in red. The number of deleted nucleotides are shown to the right of each allele.

Thyroid stimulating hormone receptor (TSHR), activated by its ligand thyroid stimulating hormone (TSH, aka thyrotropin), plays essential roles in the differentiation, growth and function of thyroid^1^. Human TSHR, encoded by the *TSHR* gene consisting of 10 exons on chromosome 14q31, is a G-protein-coupled receptor (GPCR) with one extracellular domain (ECD), seven transmembrane domains (TMDs), three extracellular loops (ECLs), three intracellular loops (ICLs), and one intracellular carboxy terminal (Fig. 1A). ECD is encoded by the first 9 exons and part of exon 10, which has a leucine-rich region (LRR) for ligand binding. TMDs and ICLs, encoded by the exon 10, are responsible for activating G-protein-coupled effectors, adenylatecyclase (AC) and phospholipase C (PLC)^2^.

Congenital hypothyroidism (CH) is a common endocrine metabolic disease which is caused by insufficient thyroid hormone secretion at birth or in utero. Generally, the prevalence of CH is around 1 in 3,000–4,000 globally^3,4^, and the incidence has increased significantly in the recent years^1,5^. CH differs in etiopathogenesis and clinical symptoms. Loss-of-function (LOF) mutations in *TSHR* are found most frequently in TSH resistance leading to CH^6^, which is characterized by high TSH serum level, normal or reduced thyroid hormone concentrations, normal sized or hypoplastic thyroid gland^1^. To date, there are less than 10 cases reporting nonsense and/or frame-shift mutations in the exon 10 of *TSHR*^7–9^. Interestingly, homozygous nonsense mutation (1431C > G) in the exon 10 was found in a female patient, which results in the truncated TSHR with only ECD, TMD1 and ICL1 domains^10^. However the molecular mechanism is not yet investigated.

Genetic CH animal models have relatively stable phenotypes without surgical trauma and are technically easy to handle and maintain^11^. However, for decades, there are only a few TSH-resistance CH mouse models available, e.g., spontaneous mutation stains *Tshr*^*hyt/J*^^12^, *Tshr*^*hyt−2J/GrsrJ*^, and *Tshr*^*hyt−3J/GrsrJ*^, and a null mutation strain *Tshr*^*tm1Rmar/J*^^13^. Rat is bigger in size than mouse so that to provide larger amount of blood/tissue samples for accurate assessment. Moreover, rat is more physiologically similar to human^14^ and thus shows similar phenotypes to those in human which sometimes are not seen in the mouse counterpart^15^. Taking advantage of the CRISPR gene-editing technology, we sought to develop a novel rat model to functionally characterize the truncated Tshr protein *in vivo*.

In this study, we generated several *Tshr* mutant rats after zygotic injection of Cas9 mRNA and sgRNAs targeting the exon 10 of *Tshr*. We particularly studied a rat strain (designated as *Tshr*^*Df/Df*^) carrying homozygous alleles of 5-bp deletion, which results in truncated Tshr protein missing all the domains after ICL1. *Tshr*^*Df/Df*^ showed thyroid hypoplasia with TSH resistance and low levels of both free triiodothyronine (T3) and free thyroxine (T4). The hypothyroidism phenotypes are confirmed to be related to the suppression of thyroid specific genes, such as thyroperoxidase (*Tpo*), thyroglobulin (*Tg*), and sodium-iodide symporter (*Nis*). In conclusion, we created a novel rat model by truncating Tshr, which will serve as a useful tool to dissect the function of Tshr during development and the pathomechanism of CH in the future.

## Results

### **Generation of*Tshr*mutant rats**

Protein sequence alignment showed that Tshr is revolutionarily conserved in rat and human (HomoloGene in NCBI). To verify the predicted transcripts in NCBI website, we extracted the total RNA from thyroid glands of 3 wild-type (WT) SD rats and designed specific PCR primers to distinguish them. The RT-PCR results indicated that only the transcript NM_012888.1 exists in the rat thyroid (Supplementary Fig. 1). We created *Tshr* mutant rats by CRISPR/Cas9 gene editing tool following the protocol as previously reported^15^. Briefly, we designed two specific targeting sequences in the 10th exon (Fig. 1B), which were subsequently cloned into pX330 vector respectively. Using *in-vitro* T7EI assay, we verified that both targeting sequences can lead to DNA double strand break and subsequent NHEJ (Fig. 1C). So we arbitrarily selected the construct Tshr1 for microinjection. Cas9 mRNA and sgRNA, both at a concentration of 25 ng/μL, were injected into the zygotes of SD rats. After oviduct implantation, we obtained 13 founders from total 60 transferred embryos (Table 1). Interestingly, genotyping showed that all the founders are compound heterozygous. We did not even detect any mosaicism using the DNA extracted from the tail. As summarized in Fig. 1D, we obtained five alleles which carry different deletions and/or insertions near the gRNA targeting site due to NHEJ. In order to evaluate off-target effect (OTE), we predicted the potential sites by GT-Scan^16^. After examining 5 sites which OTE will most likely occur (Supplementary Table 1), we found no detectable OTE.

**Table 1:** Generation of *Tshr* mutant rats via the CRISPR/Cas system.

Pseudo- pregnancy	Embryos transferred	Newborn	Mutant		Compound heterozygous	Homozygous	Mosaic
			Male	Female			
1	20	6	3	3	6	0	0
2	20	7	3	4	7	0	0
3	20	0	0	0	0	0	0

### Impaired thymus development in the *Tshr*^*Df/Df*^ rats

**Figure 2:**
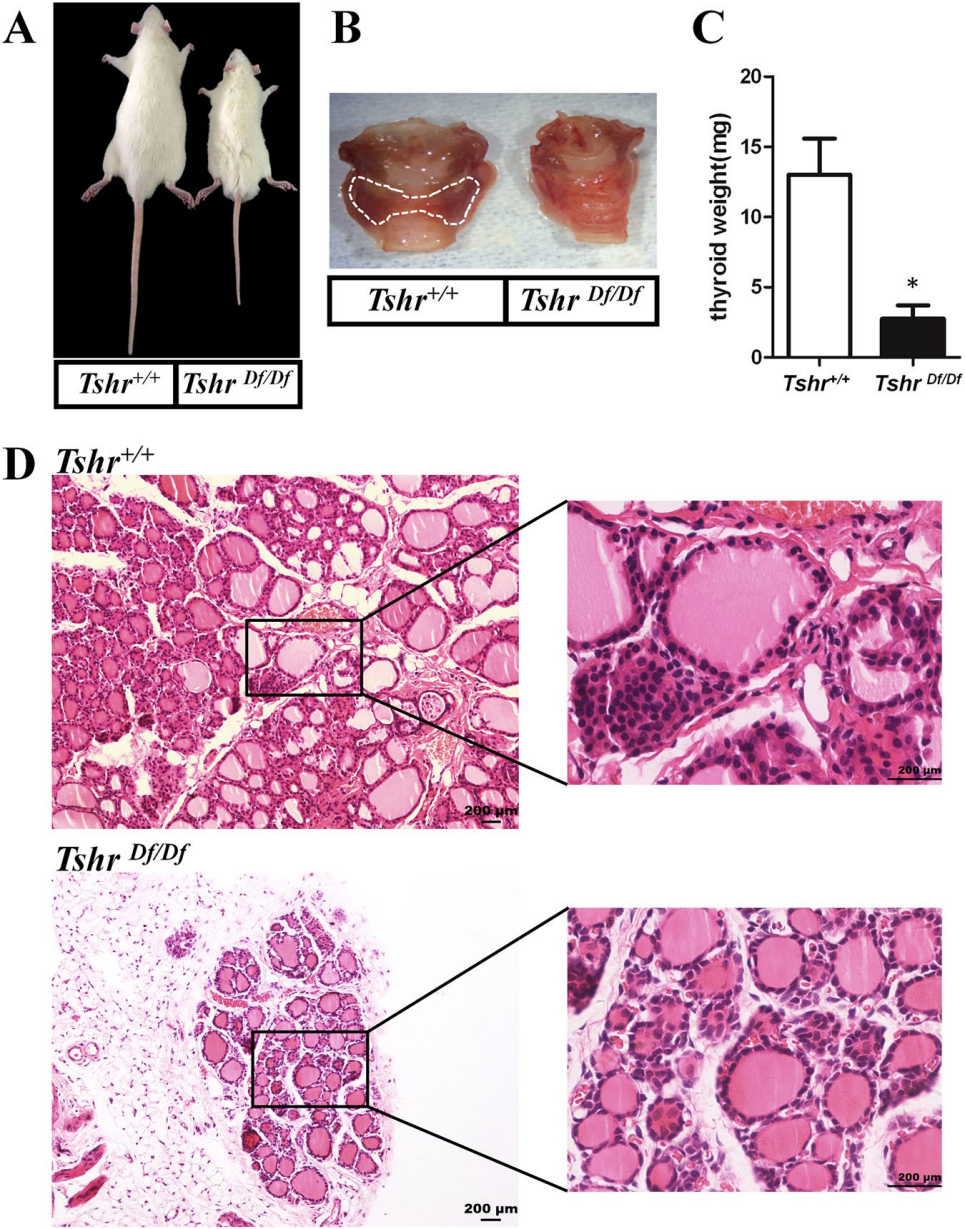
Morphological examination of thyroid revealed the phenotypes of congenital hypothyroidism in *Tshr*^*Df/Df*^ rats. (**A**) A male *Tshr*^*Df/Df*^ rat showed dwarf phenotype at age of 8 weeks compared to a male WT rat. (**B**) The thyroid glands of *Tshr*^*Df/Df*^ rats are very small and not visible in comparison to the WT littermates (Thyroid glands are circled in dash line). The phenotype was observed at week 8. (**C**) The wet thyroid weight in *Tshr*^*Df/Df*^ rats (n = 4) at week 8 is significantly smaller than that of the WT littermates (n = 4). Error bar indicates standard deviation of the mean. *p < 0.01. (**D**) HE staining showed decreased number of thyroid follicular cells and parafollicular cells in the *Tshr*^*Df/Df*^ rats in comparison to the WT littermates. Thyroid follicular cells in the *Tshr*^*Df/Df*^ rats are inactive with flat shaped cytoplasm.

As stated above, the founders are compound heterozygous, showing the dwarf phenotype as early as 3-week old. To circumvent the infertility problem, we treated the founders with levothyroxine sodium every day by i.g. administration which allowed germline transmission. Among the progeny, we characterized the homozygous mutant rats with deletion of CACGC (designated as *Tshr*^*Df/Df*^) and used them for the following experiments. This five-nucleotide deletion results in frameshift that introduces an early stop codon at the position 478 of the wild-type Tshr protein (Supplementary Fig. 2). We found that the thyroids in *Tshr*^*Df/Df*^ rats are barely observed at the age of 8 weeks (Fig. 2B), and their weight is significantly smaller than those in the WT controls (Fig. 2C). We did not observe any phenotypes in the heterozygotes (data not shown), which is in line with the findings in the other hypothyroidism mouse models. The HE staining showed that there is a marked decrease in the number of both thyroid follicular cells and parafollicular cells in the *Tshr*^*Df/Df*^ rats (Fig. 2D). In addition, thyroid follicular cells in the *Tshr*^*Df/Df*^ rats are flat shaped with thin cytoplasm, while those in the control *Tshr*^+/+^ rats are cuboidal or columnar with thicker cytoplasm (Fig. 2D). This indicated that thyroid follicular cells in the *Tshr*^*Df/Df*^ rats are not functionally active in producing the thyroid hormones T3 and T4.

### Changes in serum thyroid hormone level and body weight

**Figure 3:**
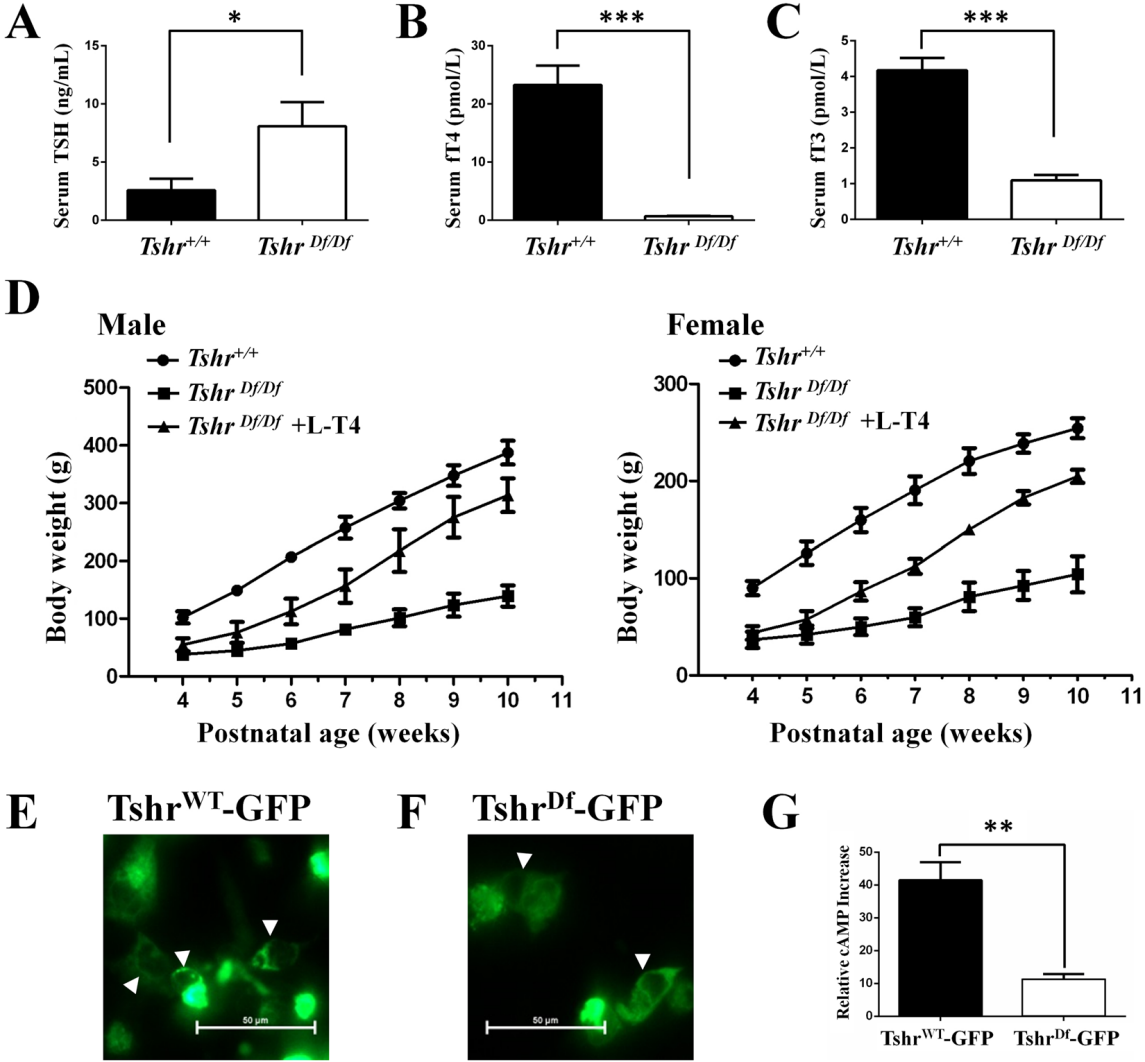
Endocrinological trait and body weight of the *Tshr*^*Df/Df*^ rats are resulted from low cAMP induction by truncated Tshr. (**A–C**) Thyroid-stimulating hormone (TSH), free thyroxine (fT4) and free triiodothyronine (fT3) levels were determined by CLIA respectively in the *Tshr*^*Df/Df*^ rats at the age of 8 weeks. Data are shown in Mean ± SD (In TSH assay, n = 4 for the *Tshr*^*Df/Df*^ rats and their WT littermates; In fT4 and fT3 assays, n = 4 for the *Tshr*^*Df/Df*^ rats and n = 6 for the WT littermates). (**D**) Comparison of the body weight of the *Tshr*^*Df/Df*^ rats with/without L-T4 treatment to their WT littermates in both male and female respectively. In males, n = 4 for the *Tshr*^*Df/Df*^ rats, n = 5 for the WT littermates, and n = 3 for the *Tshr*^*Df/Df*^ rats treated with L-T4 from 4 to 10 weeks of age. In females, n = 4 for the *Tshr*^*Df/Df*^ rats, n = 6 for the WT littermates, and n = 3 for the *Tshr*^*Df/Df*^ rats treated with L-T4 from 4 to 10 weeks of age. The p values are in Supplementary Table 4. (E, F) GFP-fused wild-type Tshr (**E**) and truncated Tshr (**F**) both locate on the cell-membrane (arrow heads) of transfected Cos7 cells. (**G**) Truncated Tshr leads to significantly low cAMP level after TSH stimulation. The cAMP level was normalized with that of pAAV-GFP transfected cells. Data are presented as the Mean ± SD (n = 3). *p < 0.01; **p < 0.001; ***p < 0.0001.

We next examined the serum hormone levels in the *Tshr*^*Df/Df*^ rats at the age of 8 weeks. We found that the serum TSH level is significantly higher in the *Tshr*^*Df/Df*^ rats than in the WT controls most likely due to the feedback loop (Fig. 3A). The *Tshr*^*Df/Df*^ rats have hypothyroidism with significant decrease in the serum levels of both free triiodothyronine (fT3) and thyroxine (fT4) (Fig. 3B,C). As a result, the *Tshr*^*Df/Df*^ rats grow slowly and can be easily distinguished from the WT or heterozygous littermates at 2 weeks old for their small sizes (Fig. 3D). Without the thyroid hormone treatment, the *Tshr*^*Df/Df*^ rats will carry the dwarf phenotypes throughout their lifespan. After oral administration of levothyroxine sodium (L-T4) at 4 weeks old, both male and female *Tshr*^*Df/Df*^ rats have a significant gain of body weight since week 5, although they still cannot reach the same body weight as that of the WT males and females respectively (Fig. 3D, Supplementary Table 2). This phenotype is similar to that of *hyt/hyt* mice^12^. The levothyroxine treatment also rescues the infertility phenotype of the *Tshr*^*Df/Df*^ rats. All the untreated *Tshr*^*Df/Df*^ rats are infertile and some die early because of weakness (data not shown). To investigate how the truncated Tshr is functionally impaired, we cloned the endogenous WT *Tshr* and truncated *Tshr* cDNA into pAcGFP1-N1 vector respectively. After we transfected Cos7 cells with these constructs, we found that the truncated TSHR protein localizes on the cell-membrane as does the WT TSHR (Fig. 3E,F). We then stimulated the transfected cells with human TSH and found that the cAMP level induced by the truncated TSHR is significantly lower than that induced by WT TSHR (Fig. 3G). Taken together, missing six transmembrane domains does not affect TSHR’s membrane localization, but rather reduces its ability of activating the G protein to increase intracellular cAMP level.

### The altered expression of thyroid specific genes

**Figure 4:**
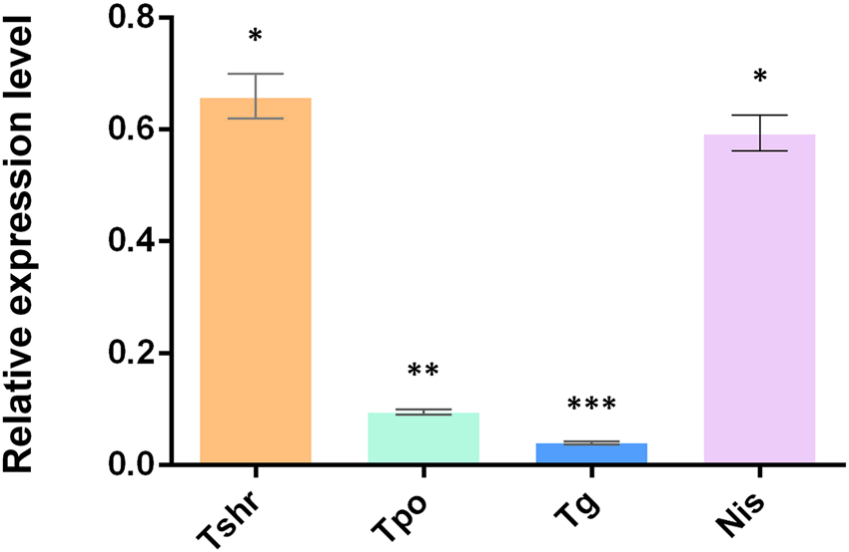
The thyroid specific genes are suppressed at the mRNA level in the *Tshr*^*Df/Df*^ rats. The expression of truncated TSHR only reaches about 60% of that of the WT controls. The thyroid specific genes, i.e., *Tpo*, *Tg* and *Nis*, are significantly suppressed in the *Tshr*^*Df/Df*^ rats. Data are presented as the Mean ± SEM (n = 3). *p < 0.05; **p < 0.01; ***p < 0.001.

**Figure 5:**
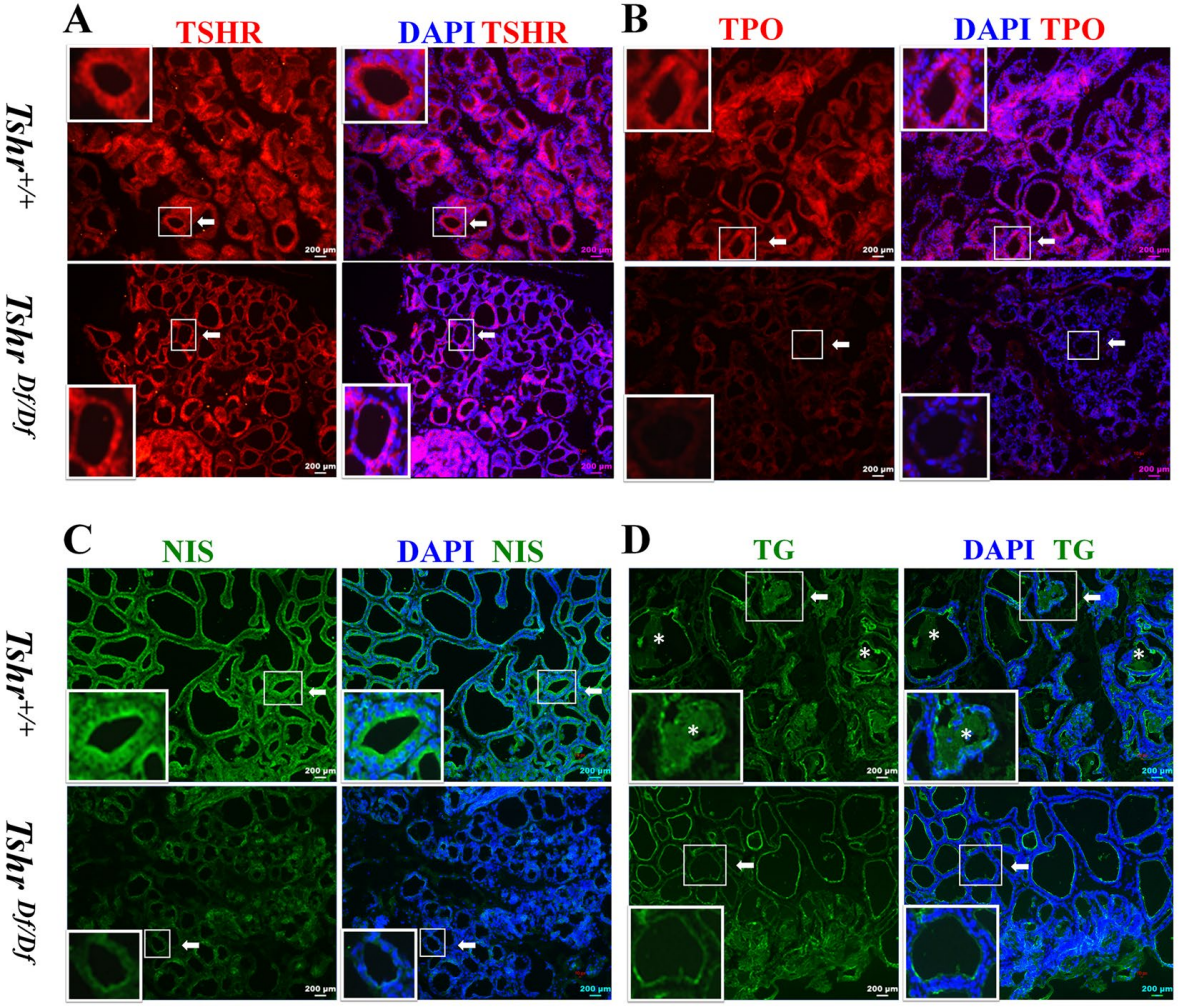
Immunofluorescence showed expression alteration of thyroid specific genes. (**A**) The truncated TSHR protein is expressed in the thyroid follicular cells. Please note that the thyroid follicular cells display a flat shape in the *Tshr*^*Df/Df*^ rats, while those in the WT controls are active with cuboidal or columnar shape. (**B–D**) The expression level of TPO, NIS and TG is significantly lower in the *Tshr*^*Df/Df*^ rats than the WT controls. The asterisks in (**D**) indicate the presence of TG protein in colloid in thyroid follicles of the WT controls.

As *Tshr* mutation in mice affect the expression of other thyroid specific genes^13,17^, we sought to examine some of these genes essential for thyroid development. We first checked the expression of *Tshr* itself by real-time RT-PCR with two different primer sets. Interestingly, we found that the truncated *Tshr* transcription only reaches average 66% of that in the WT controls (Fig. 4). The lower expression of mutant Tshr protein is also confirmed by immunofluorescence using polyclonal antibody that recognizes N terminal of TSHR protein (Fig. 5A). As stated above, the thyroid follicular cells in the *Tshr*^*Df/Df*^ rats are flat, while those in the control *Tshr*^+/+^ rats are cuboidal or columnar shaped (Fig. 5A). We found that the expression of *Tpo* and *Nis* is significantly suppressed in the *Tshr*^*Df/Df*^ rats at both mRNA (average 9% and 66% of that in the WT controls respectively, Fig. 4) and protein (Fig. 5B,C) level, which is similar to what is observed in *Tshr* mutant mice^13,17^. Interestingly, real-time RT-PCR showed that the *Tg* transcription is significantly suppressed in the thyroid of the *Tshr*^*Df/Df*^ rats (average 4% of that in the WT controls, Fig. 4). Consistently, the abundance of TG protein, especially in the colloid, is also decreased in the *Tshr*^*Df/Df*^ rats (Fig. 5D).

## Discussion

We successfully created the CH rat model *Tshr*^*Df/Df*^ by CRISPR/Cas9 technology. This is the first time as we know that frameshift and/or nonsense mutation in human *TSHR* is modeled in a mammalian system at whole organism level. Consistent with what was observed in the human recessive nonsense mutation Y444X^10^, the *Tshr*^*Df/Df*^ rat is characterized as thyroid hypoplasia with slow growth rate, small thyroid gland, elevated TSH and low levels of both free T3 and free T4. TSHR Y444X patient received L-T4 treatment since the 10^th^ day of life and were physically normal afterwards^10^, however, *Tshr* null mice will die within a week after weaning without L-T4 treatment^13^. Interestingly, most of the *Tshr*^*Df/Df*^ rats can live a normal life span without the L-T4 treatment (data not shown), which implied that their phenotypes are less severe than the null mutants. Since this mutation is not lethal, out results suggested that there may exist many other frameshift and/or nonsense mutations in human *TSHR* at similar locations in exon 10.

Phenotypes in different *Tshr* mutant animal models may vary likely due to different genetic alterations, which should be considered when choosing the proper animal model for specific research area. As stated previously, *Tshr*^*hyt/J*^^12^, *Tshr*^*hyt−2J/GrsrJ*^, and *Tshr*^*hyt−3J/GrsrJ*^ mice are spontaneous mutations, while *Tshr*^*tm1Rmar/J*^ carries a null mutation created by disruption of the gene with an EGFP insertion^17^. *Tshr*^*hyt/J*^ is the most widely used “*hyt/hyt*” mouse which carries C to T transition causing L556P in TMD4. *Tshr*^*hyt−2J/GrsrJ*^ has C to T transition at a different position (Chr12: 91,537,844), which leads to a stop codon in TMD3 (www.jax.org). *Tshr*^*hyt−3J/GrsrJ*^ has a 232-nucleotide deletion, which leads to frame shift after amino acid 442 (S442Mfs*10, www.jax.org). *Tshr*^*tm1Rmar/J*^ certainly has the most severe phenotype because the homozygotes die in one week after weaning (at day 21) and its TSH level is 400 fold higher than the wild type^17^. By contrast, TSH level in *Tshr*^*hyt/J*^ is only 100 fold higher than that in the wild type^11^. *Tshr*^*hyt−2J/GrsrJ*^ and *Tshr*^*hyt−3J/GrsrJ*^ are not well characterized compared to hyt/hyt mice, however they may have different pathological mechanism in hypothyroidism. Take reproduction for example, survived adult *Tshr*^*hyt−3J/GrsrJ*^ have reduced fertility but are not sterile (www.jax.org). However, all the other *Tshr* mouse strains and our *Tshr*^*Df/Df*^ rats were reported to be sterile. Taken together, it implies that the particular mutation in *Tshr*^*Df/Df*^ rats probably regulates thyroid hormone levels in a different way which should be further studied in the future.

In the *Tshr*^*Df/Df*^ rats, both transcription (Fig. 4) and translation levels (Fig. 5) of *Tg* are lower than the WT controls at 8 weeks of age. Colloid in the thyroid follicles appears normal in the *Tshr*^*Df/Df*^ rats (Fig. 2D), which is different from the phenotype of lacking colloid in *Tpo* mutant mice^18^. So it is likely that the truncated TSHR results in decreased cellular level of cyclic adenosine monophosphate (cAMP) that is required for rapid exocytosis of TG^19^, thus accumulated TG protein in the follicular cells suppresses its own expression by a negative feedback mechanism. In contrast, *Tg* expression was found to be unchanged in *hyt/hyt* and *Tshr* null mice^13,17^, except that its iodination relies on TSH signaling^13^. One possible explanation is that these studies used low-resolution methods, such as western blot and immunohistochemistry, which cannot precisely distinguish the changes of *Tg* expression level. We also cannot exclude the variation due to genetic difference between mouse and rat. Nevertheless, plasma TG level in the *TSHR* Y444X patient is relatively low compared to the healthy family members, although at the normal range^10^. Taken together, our *Tshr*^*Df/Df*^ rat is a good model which recapitulates the symptoms in human CH disease carrying TSHR Y444X mutation.

The creation of *Tshr*^*Df/Df*^ rats also serves as another example of modeling the human genetic disorders resulted from frameshift and/or nonsense mutations in certain gene(s) by CRISPR/Cas9 system. More than 10 loci of frameshift and/or nonsense mutations in *TSHR* have been reported in different families^10,12,20–35^. However, none of these mutations have been functionally characterized, particularly in an animal model. For example, Y444X mutation has not been investigated either *in vitro* or *in vivo* before we created the *Tshr*^*Df/Df*^ rats. Meanwhile, many TSHR missense mutations have been reported in human diseases, which are of great importance for studying the GPCR mediated signaling transduction. For example, P556R mutation was characterized as thyroid aplasia in a family of Turkish origin^36^. The only related animal model is *hyt/hyt* mouse, in which the replacement of Pro with Leu (P556L) in TMD4 of *Tshr*^12^ abolishes the binding of TSH to TSHR^37^, leading to reduction of cAMP and thyroid hormones^11,38^. Of note, previous studies usually studied the functional domains and their interactions by substitution of individual amino acids *in vitro*^39^. However, the output of GPCR signaling that is analyzed in different *in-vitro* systems may not be consistent^40^. So the *in-vivo* validation is one of the most important analyses to functionally characterize the essential amino acids in GPCR (e.g., TSHR). With the fast development of CRISPR/Cas technology, especially its application in homologous recombination, we expect that the function of each essential amino acid in GPCR will be validated in the future using animal model systems in addition to X-ray crystallography.

The *Tshr*^*Df/Df*^ rat offers a versatile tool for studying the developmental and functional abnormalities in different tissues/organs due to the reduction of thyroid hormones, which are not fully explored in this study. Hypothyroidism causes abnormalities in brain including altered neuronal growth and maintenance, synaptogenesis, and myelination, etc. In *hyt/hyt* mice, for example, certain tubulin isoforms such as Mβ5 and Mβ2 are down regulated in layer V pyramidal neurons in the sensorimotor cortex^11^. Since rat has more sophisticated behaviors than mouse, the *Tshr*^*Df/Df*^ rats will be useful to dissect the subtle neurological disorders. We have not tested whether the *Tshr*^*Df/Df*^ rat is congenitally deaf as the *hyt/hyt* mice^41–43^. Moreover, we are interested in the molecular etiology of infertility in both male and female *Tshr*^*Df/Df*^ rats. In line with the previous findings in *hyt/hyt* mice^12^, we found that both male and female *Tshr*^*Df/Df*^ rats are infertile but can be reversed by L-T4 treatment after weaning. However, the onset of infertility, genes and pathways that are altered during sexual maturation are still needed to be addressed in details. In conclusion, we generated and characterized a novel rat model that recapitulates the phenotypes in TSHR Y444X mutation in human, which will also help to study GPCR related signaling during development and the pathomechanism of CH.

## Materials and Methods

### Animal care

Wild type Sprague-Dawley (SD) rats were purchased from Shanghai SLAC Laboratory Animal Co Ltd. Rats were kept in the Animal Center of Tongji University under controlled temperature, humidity and light. Standard food and water were supplied *ad libitum*. All the experimental procedures as described below were performed in accordance with the guidances and regulations approved by the animal experiment administration committee of Tongji University (TJLAC-015–029).

### Generation of *Tshr* knockout rats by CRISPR/Cas9 genome editing technique

We generated *Tshr* knockout rats by CRISPR/Cas9 genome editing technique exactly following our previous protocol^15^, including *in-vitro* transcription of Cas9 mRNA and gRNAs, intracytoplasmic RNA microinjection, and genotyping. The detailed information of the oligoes/primers is in Supplementary Table 3.

### Off-target analysis

The potential off-target sites were predicted by GT-Scan^16^, among which the top 5 ranked sites were selected. Primers were designed to amplify ~700 bp DNA fragment flanking each off-target sites (Supplementary Table 1). PCR was done with Q5 High-Fidelity DNA Polymerase (NEB): 98 °C for 30 S; 35 cycles of 98 °C for 10 s, 58 °C for 15 s, 72 °C for 20 s; and 72 °C for 2 min. The PCR products were subcloned by Lethal Based Fast Cloning Kit (Tiangen Biotech). Ten positive colonies were picked for Sanger sequencing.

### Oral administration of levothyroxine sodium

Levothyroxine sodium was dissolved in the 50 mM NaOH with a working concentration of 4 ug/ml, aliquoted and stored at −20 °C. Levothyroxine sodium solution was vigorously mixed before it was given to the weaned rats (from the age of week 4) by intragastric administration (i.g.) (0.04 μg/g).

### Histological analysis of thyroid gland

The thyroid gland was fixed in 4% paraformaldehyde for 1 week and embedded in paraffin. The sections (5 μm) were mounted on glass slides and counterstained with hematoxylin and eosin (H&E). All the slides were examined and digital images were taken on Nikon ECLIPSE Ti.

### Measurement of serum TSH, fT3 and fT4

Blood collected from the tail vein of the rats was chilled on 4 °C overnight and then spun at 6000 g for 10 min before collection of the serum. TSH, fT3 and fT4 levels in the serum were measured by chemiluminescence immunoassay (CLIA) respectively (Rat TSH CLIA Kit (E-CL-R0647) was purchased from Elabscience. The serum fT3 and fT4 levels were examined by Adicon Central Lab.).

### Real-time RT-PCR

Total RNA was isolated from the dissected thyroids by Quick-RNA Micro Prep Kit (Qiagen). cDNA was prepared by RevertAid First Strand cDNA Synthesis Kit (Thermo). Real-time PCR was subsequently performed by QuantiNova^TM^ SYBR Green PCR Kit (Qiagen) in the ABI 7900 Fast Real-Time PCR System. The detailed primer information is in Supplementary Table 4. *Gapdh* was used as internal control. Fold change in gene expression was calculated by following the comparative CT method (ΔΔCT method) in ABI’s protocol.

### Immunofluorescence

Thyroid glands from 10-week-old rats were placed in 4% PFA overnight. They were dehydrated in 30% sucrose solution and then placed in OCT at 4 °C overnight. Serial sections of 10 μm thickness were dried on a clean bench for 10 minutes. Then they were washed with PBS for 3 times followed by incubation in 4% PFA for 30 minutes. Then the slides were incubated in the boiling antigen repair solution (0.01 M citrate buffer (pH 6.0, 3 g of trisodium citrate and 0.4 g of citric acid in 1 L ddH_2_O) for 10 minutes and cooled down to room temperature. The slides were washed with PBS for 3 times, and then incubated with the primary antibody (TPO (Abcam ab203057), TSHR (ABclonal A6781), NIS (ABclonal A9605) and TG (Abcam ab156008); 1:200 in PBS-TB buffer (PBS with 5% Triton, 5% goat serum and 2% BSA) for 24 hours. After washed with PBS for 5 times, the slides were incubated with both the secondary antibody and DAPI solution (1:400) at room temperature for 1 hour. Finally the slides were washed with PBS for 5 times and sealed for fluorescence detection by Nikon ECLIPSE 80i.

### Cell transfection and cAMP detection

Cos7 cells were cultured in DMEM as routine. The cells were seeded in a 6-well plate (Corning) and transfected with the constructs of rat wild-type Tshr (*Tshr*^*WT*^*-GFP*) and truncated Tshr (*Tshr*^*Df*^*-GFP*) in pAcGFP1-N1 (Clontech, 632469) backbone (The cells transfected with GFP expression vector pAAV-GFP was used as negative control). The RT-PCR primers for cloning the gene are as follows: For *Tshr*^*WT*^-GFP, WT-F: 5′-CTACCGGACTCAGATCTCGAGCCACCATGAGGCCAGGGTCCCTGCTCC-3′; WT-R: 5′-CGACTGCAGAATTCGAAGCTTCAGGGCTGTTTGCGTGTACTCTTCT-3′; For *Tshr*^*Df*^-*GFP*, WT-F: 5′-CTACCGGACTCAGATCTCGAGCCACCATGAGGCCAGGGTCCCTGCTCC-3′; MUT-R: 5′-CGACTGCAGAATTCGAAGCTTGTGCACGGTCAGTTTGTAGTGGCTAG-3′. Two days post transfection by Lipo3000 (Invitrogen), GFP signal was detected and recorded by Nikon ECLIPSE Ti. Then the cells were stimulated by human TSH (YuduoBio) for 1 h before cAMP in the cell lysate was determined by Mouse/Rat cAMP Assay (R&D, KGE012B) kit following the manufacturer’s protocol.

## Electronic supplementary material

Supplementary Data
