# Supplementary material for: Generation and characterization of a hypothyroidism rat model with truncated thyroid stimulating hormone receptor
Source: Sci Rep. 2018 Mar 5;8:4004. doi: 10.1038/s41598-018-22405-7 (PMC5838214; doi:10.1038/s41598-018-22405-7)
Supplement: Supplementary file 1 — Supplementary Data [file 41598_2018_22405_MOESM1_ESM.doc]

**Supplementary Data**

**Generation and characterization of a hypothyroidism rat model with truncated thyroid stimulating hormone receptor**

Jianqiang Yang^1^*, Ning Yi^2^*, Junhui Zhang^2^, Wen He^2^, Di He^3^, Wanwan Wu^3^, Shuyang Xu^3^, Feng Li^4^, Guoping Fan^5^, Xianmin Zhu^3#^, Zhigang Xue^2#^, Wensheng Zhou^1#^

*These authors contributed equally to this work.

^#^Correspondence should be addressed to W Zhou, Z Xue or X Zhu, Email: zhouwesh@163.com, xuezg@tongji.edu.cn or xianminzhu@hotmail.com.


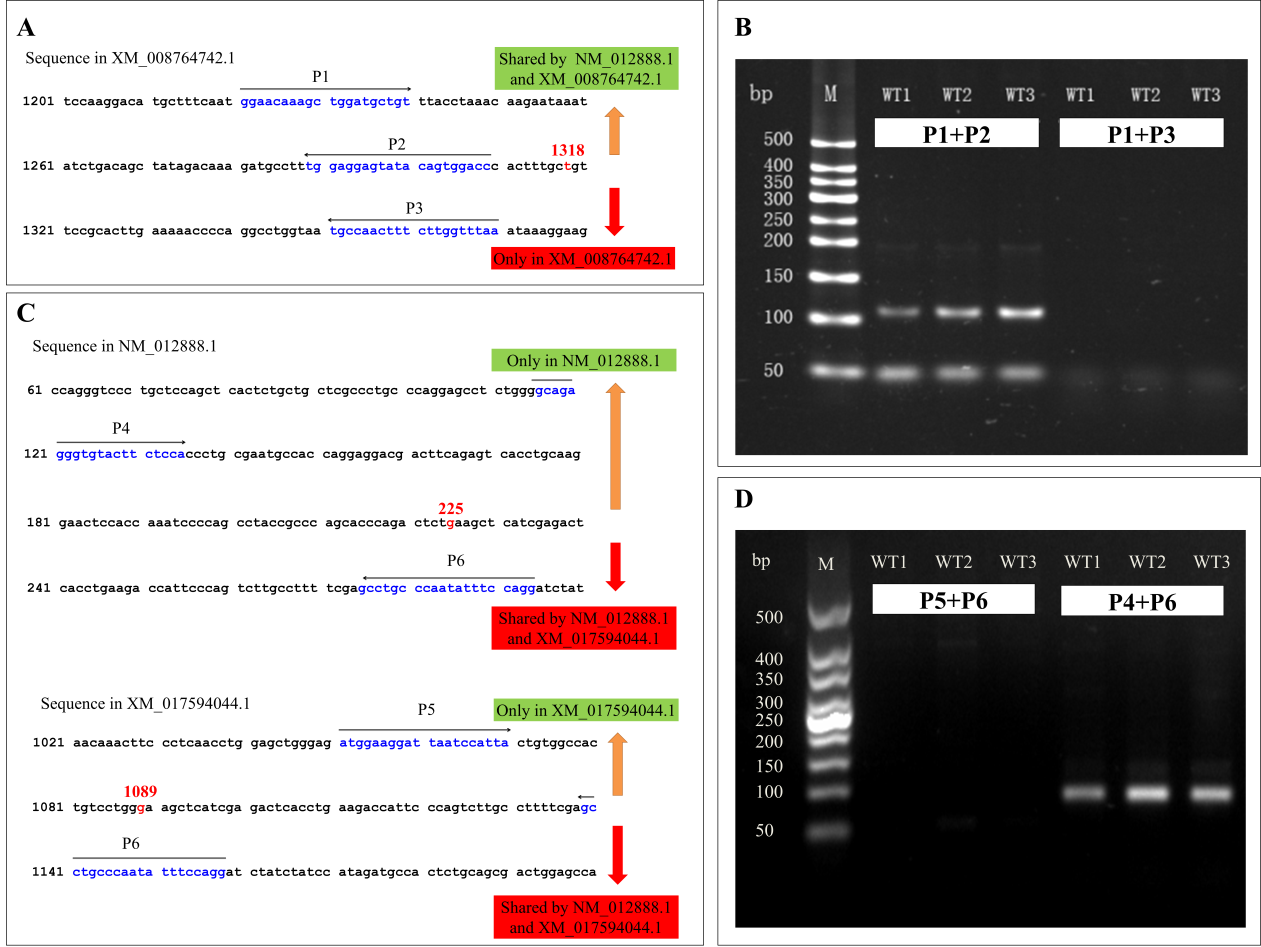


**Supplementary Figure 1. The RT-PCR results indicated that only the transcript NM_012888.1 exists in the rat thyroid.** (A) XM_008764742.1 encodes a truncated version of NM_012888.1, which shares the 5’UTR and the sequences from ATG to the T (in red) at position 1318. Primers 1, 2 and 3 (sequence in blue) are designed to distinguish XM_008764742.1 and NM_012888.1. (B) After RT-PCR of the RNA from thyroid of 3 WT SD rats (WT1, WT2, and WT3), PCR products can be only detected in reactions using primers P1 and P2, not in those using P1 and P3. So the mRNA isoform with the sequence as XM_008764742.1 does not exist in SD rat thyroid.(C) NM_012888.1 and XM_017594044.1 share the DNA sequence after the position 225 (G in red) and the position 1089 (G in red) respectively. Primers 4, 5 and 6 (sequence in blue) can distinguish the different 5’ sequences in NM_012888.1 and XM_017594044.1. (D) After RT-PCR of the RNA from thyroid of 3 WT SD rats (WT1, WT2, and WT3), PCR products were only detected using primers P4 and P6. So the mRNA isoform with the sequence as XM_017594044.1 does not exist in SD rat thyroid.


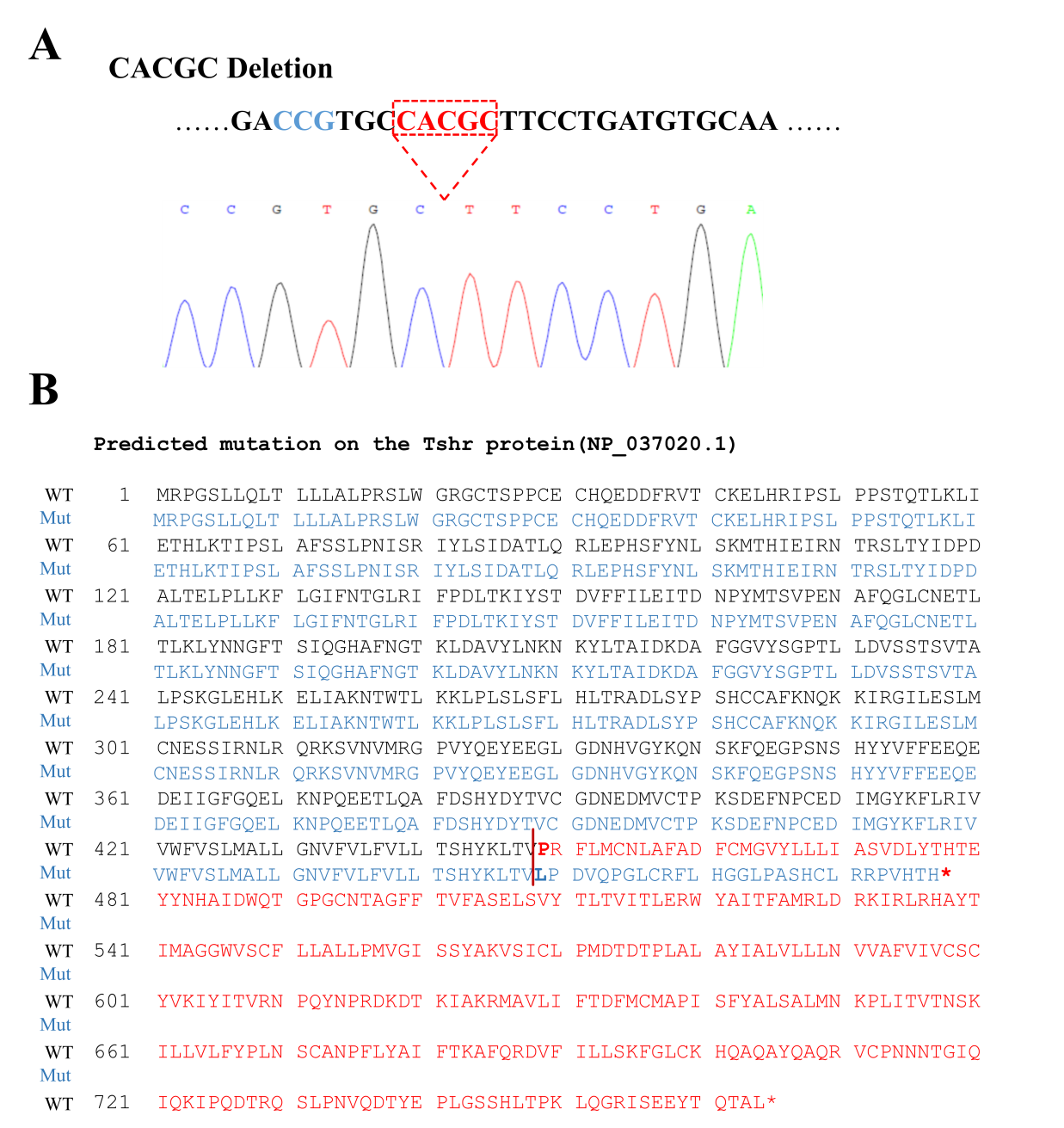


**Supplementary Figure 2. The frameshift and nonsense mutation in the *Tshr^Df/Df^* rats.** (A) The 5-bp deletion (CACGC) in exon 10 is indicated in red. Below is the representative DNA sequence chromatogram. (B) The deletion of CACGC is predicted to introduce frameshift at residue 449 and a stop codon at 478 (P449fsX478).


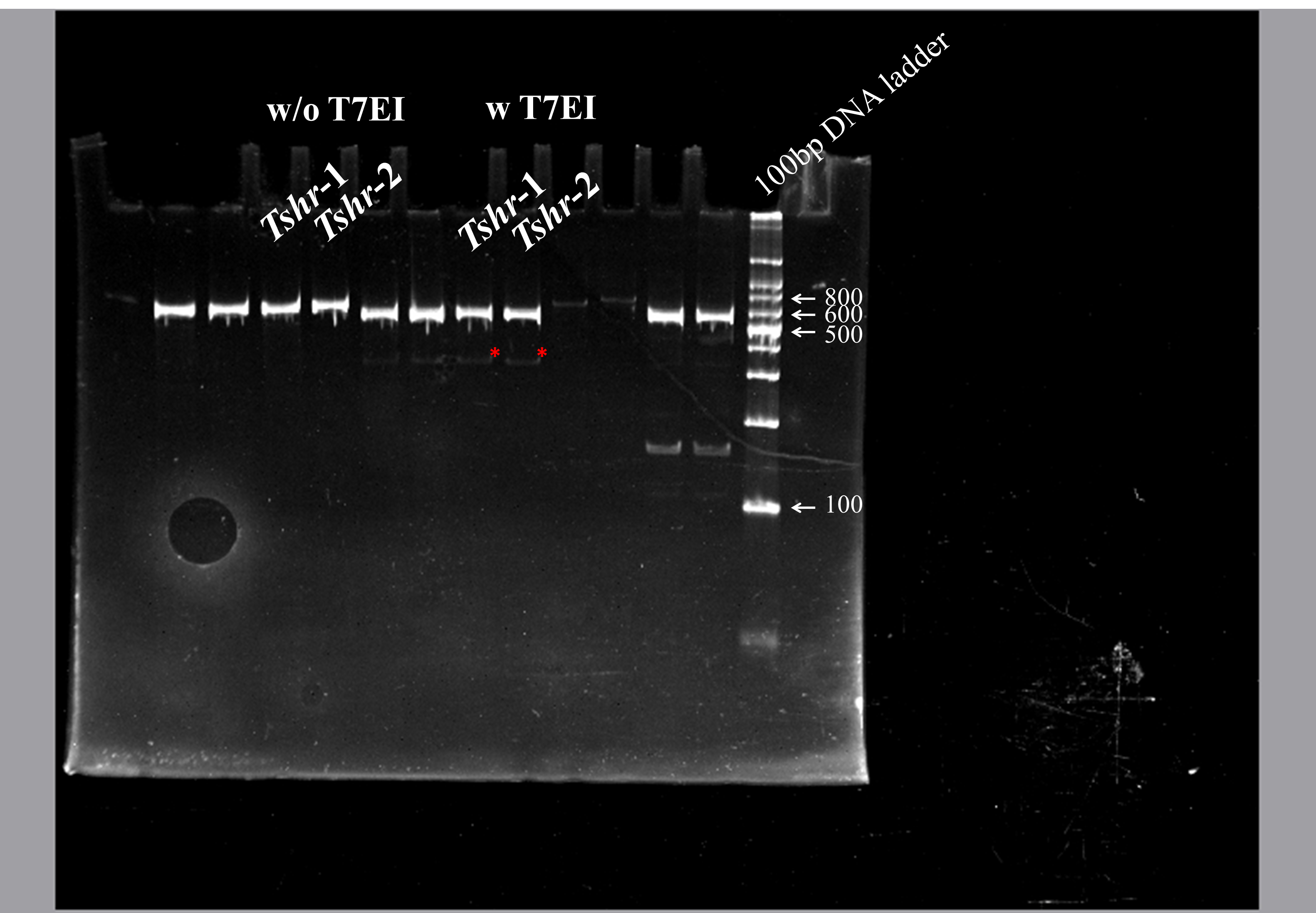


**Supplementary Figure 3. The original gel picture of Figure 1C.**

**Supplementary Table 1**. The potential off-target sites and their detection primers.

| **Potential off-target site** | **Sequence** | **Primers for detection** |
| --- | --- | --- |
| *Tshr* targeting sequence | CCGTGCCACGCTTCCTGATGTGC |  |
| Chr5-1, Position 120255630 | CCCTGCCCCTCTTCCTGATGTGC | C51-F: TTGCAGAACCCCTCAAAAGC |
|  |  | C51-R: CCAGACACAGTGCGATGAAC |
| Chr5-2, Position 164555572 | CACTGCCAGGCTTCCTGATGTGC | C52-F：AGCACTGACTTGGGAGATCT |
|  |  | C52-R：CACAGCCTGCTTGACCTT |
| Chr4-1, Position 59476811 | ATGTGCCACGCTTCCAGATGTGC | C41-F：GGGAACACGTACTCTGGGAT |
|  |  | C41-R：ACTTGGGGACTCACTCAACC |
| Chr4-2, Position 62240754 | CCATGCCACTCTTCCTGATTTAC | C42-F：GGGAACACGTACTCTGGGAT |
|  |  | C42-R：ATCTCTGGCCAAAAGGAGCT |
| Chr10, Position 10600516 | CACTGCCATGCTTCCTGATGTGA | C10-F：GTGCAGTATCATGCTCAGCC |
|  |  | C10-R：TTTTGCTCAAGAGTGGCCAC |

**Supplementary Table 2. The p value in comparison of** **the body weight of the *Tshr^Df/Df^* rats with/without L-T4 treatment to their WT littermates.**

| **Male** | | | |
| --- | --- | --- | --- |
| **P value**  **Postnatal age** | **WT *vs* *Tshr^Df/Df^*** | **WT *vs* *Tshr^Df/Df^* +T4** | ***Tshr^Df/Df^* *vs***  ***Tshr^Df/Df^* +T4** |
| 4 weeks | | 1.364032e-005 | | --- | | | 0.000905825 | | --- | | | 0.0530095 | | --- | |
| 5 weeks | | 6.446781e-008 | | --- | | | 0.000140657 | | --- | | | 0.0216769 | | --- | |
| 6 weeks | | 2.639531e-008 | | --- | | | 0.000119617 | | --- | | | 0.00514845 | | --- | |
| 7 weeks | | 6.078872e-007 | | --- | | | 0.000905278 | | --- | | | 0.0040239 | | --- | |
| 8 weeks | | 1.095611e-007 | | --- | | | 0.00255641 | | --- | | | 0.00199659 | | --- | |
| 9 weeks | | 4.326226e-007 | | --- | | | 0.00741502 | | --- | | | 0.000731248 | | --- | |
| 10 weeks | 2.903067e-007 | 0.00538059 | 0.000184682 |
| **Female** | | | |
| **P value**  **Postnatal age** | **WT *vs* *Tshr^Df/Df^*** | **WT *vs* *Tshr^Df/Df^* +T4** | ***Tshr^Df/Df^* *vs***  ***Tshr^Df/Df^* +T4** |
| 4 weeks | | 2.378313e-006 | | --- | | | 2.785085e-005 | | --- | | | 0.251918 | | --- | |
| 5 weeks | | 1.114816e-006 | | --- | | | 2.730806e-005 | | --- | | | 0.0469147 | | --- | |
| 6 weeks | | 1.639720e-007 | | --- | | | 2.359980e-005 | | --- | | | 0.00154692 | | --- | |
| 7 weeks | | 1.138941e-007 | | --- | | | 2.964514e-005 | | --- | | | 0.000262194 | | --- | |
| 8 weeks | | 1.175258e-007 | | --- | | | 2.960533e-005 | | --- | | | 0.000306651 | | --- | |
| 9 weeks | | 2.218660e-008 | | --- | | | 2.192938e-005 | | --- | | | 0.000100763 | | --- | |
| 10 weeks | 6.295061e-008 | 6.912160e-005 | 0.000156636 |

**Supplementary Table 3. The oligoes/primers used in generation of *Tshr* mutant rats by CRISPR/Cas9 technology**.

| The oligoes for cloning gRNA sequences into PX330 vector | |
| --- | --- |
| Tshr-1F | 5’-CACCGCACATCAGGAAGCGTGGCA -3’ |
| Tshr-1R | 5’- AAACTGCCACGCTTCCTGATGTGC -3’ |
| Tshr-2F | 5’- CACCGCCTTTGCAGATTTCTGCAT -3’ |
| Tshr-2R | 5’-AAACATGCAGAAATCTGCAAAGGC -3’ |
|  | |
| The primers for verification of gRNA insertion in PX330 vector | |
| Seq F | 5'- GAGGGCCTATTTTCCCATGAT -3' |
| Seq R | 5'- GGGCGTACTTGGCATATGAT -3' |
|  | |
| The primers for *in-vitro* transcription | |
| T7-NLS-Cas9 F | 5'-TAATACGACTCACTATAGGGAGAATGGACTATAAGGACCACGAC -3' |
| T7-NLS-Cas9 R | 5'- GCGAGCTCTAGGAATTCTTAC -3' |
| sgRNA-T10-1 F | 5'- TTAATACGACTCACTATAGGTCTACCAGGAATATGAAGA -3' |
| sgRNA R | 5'- AAAAGCACCGACTCGGTGCC -3' |
|  | |
| The primers for verification of targeted mutations | |
| T10-1 F | 5'- TAGTATCCGGAACCTGCGTC -3' |
| T10-1 R | 5'- TGAAGAAACCAGCCGTGTTG -3' |

**Supplementary Table 4. The real-time RT-PCR** primers.

| **Gene** | **Primer** | **Sequence** |
| --- | --- | --- |
| *Tpo* | Tpo-L | GCTTCTGGTGGACAATGCAG |
|  | Tpo-R | ATCTCTGCTGCTCGGGAAAT |
| *Nis* | Nis-F | CTCCAGGGTTCCTTCACTG |
|  | Nis-R | CCACAGGGATACAGCCAAG |
| *Tg* | Tg-F | TCCAGACAGTTCAGTGCCAA |
|  | Tg-R | TTGTGTAGCTGGCAGAAGGA |
| *Gapdh* | Gapdh-L | CGGCAAGTTCAACGGCACAG |
|  | Gapdh-R | CGCCAGTAGACTCCACGACAT |
| *Tshr* | Tshr10.2-L | CTTTGTCCTGTTCGTCCTGC |
|  | Tshr10.2-R | TACGGAGGCAATGAGAAGCA |
